# Unraveling Prion Protein Interactions with Aptamers and Other PrP-Binding Nucleic Acids

**DOI:** 10.3390/ijms18051023

**Published:** 2017-05-17

**Authors:** Bruno Macedo, Yraima Cordeiro

**Affiliations:** Faculty of Pharmacy, Federal University of Rio de Janeiro (UFRJ), Av. Carlos Chagas Filho 373, Bloco B, Subsolo, Sala 17, Rio de Janeiro, RJ 21941-902, Brazil

**Keywords:** prion protein, nucleic acids, SELEX (Systematic Evolution of Ligands by Exponential Enrichment), aptamers

## Abstract

Transmissible spongiform encephalopathies (TSEs) are a group of neurodegenerative disorders that affect humans and other mammals. The etiologic agents common to these diseases are misfolded conformations of the prion protein (PrP). The molecular mechanisms that trigger the structural conversion of the normal cellular PrP (PrP^C^) into the pathogenic conformer (PrP^Sc^) are still poorly understood. It is proposed that a molecular cofactor would act as a catalyst, lowering the activation energy of the conversion process, therefore favoring the transition of PrP^C^ to PrP^Sc^. Several in vitro studies have described physical interactions between PrP and different classes of molecules, which might play a role in either PrP physiology or pathology. Among these molecules, nucleic acids (NAs) are highlighted as potential PrP molecular partners. In this context, the SELEX (Systematic Evolution of Ligands by Exponential Enrichment) methodology has proven extremely valuable to investigate PrP–NA interactions, due to its ability to select small nucleic acids, also termed aptamers, that bind PrP with high affinity and specificity. Aptamers are single-stranded DNA or RNA oligonucleotides that can be folded into a wide range of structures (from harpins to G-quadruplexes). They are selected from a nucleic acid pool containing a large number (10^14^–10^16^) of random sequences of the same size (~20–100 bases). Aptamers stand out because of their potential ability to bind with different affinities to distinct conformations of the same protein target. Therefore, the identification of high-affinity and selective PrP ligands may aid the development of new therapies and diagnostic tools for TSEs. This review will focus on the selection of aptamers targeted against either full-length or truncated forms of PrP, discussing the implications that result from interactions of PrP with NAs, and their potential advances in the studies of prions. We will also provide a critical evaluation, assuming the advantages and drawbacks of the SELEX (Systematic Evolution of Ligands by Exponential Enrichment) technique in the general field of amyloidogenic proteins.

## 1. Introduction

Aberrant prion proteins (PrPs) responsible for the transmissible spongiform encephalopathies (TSEs) are misfolded conformations of the natively expressed prion protein, the innocuous cellular PrP (PrP^C^) [1]. The misfolded conformers, termed scrapie PrP (PrP^Sc^), have the ability to self-perpetuate and to become infectious entities [1]. Therefore, they are the primary culprit of TSEs, which form a group of fatal neurodegenerative disorders that affect humans and other mammals [1]. Currently, the “prion” term has emerged as a new phenomenon in molecular biology, describing proteins with the ability to undergo autoconversion, autopropagation, and dissemination between cells [2]. Remarkably, pathogenic PrPs can be transmitted not only between cells but also among organisms of the same species and this can ultimately lead to epidemic outbreaks [3]. To date, only the prion protein fulfills the infectious characteristics of true prions. There is, apparently, a lack of conformational properties in other prion-like proteins to define them as bona fide prions.

PrP^C^ is a constitutive cell-surface glycoprotein, highly conserved among species, expressed in several cell types, mainly in the central nervous system (CNS) [1]. High-resolution studies have revealed two structurally distinct domains: the flexible N-terminal region (residues 23–~120) and the globular C-terminal domain (residues ~120–231), the latter composed of three α-helices and a small antiparallel β-sheet [4,5]. Is it still not known how the drastic conformational changes occur in the PrP^C^ structure—even without any mutations in the *PRNP* gene—to give rise to the abnormal PrP^Sc^. However, once formed, PrP^Sc^ can propagate in an autocatalytic manner, recruiting more PrP^C^ to fold into new PrP^Sc^, leading to its accumulation in tissues with severe cellular damage and further neurodegeneration [6]. In contrast to PrP^C^, PrP^Sc^ is a β-structure-rich protein, insoluble, and resistant to proteolysis. It can form toxic oligomers and aggregates either with an amyloid-like architecture or with an amorphous disposition [6]. Besides prion diseases, protein aggregation is the central event of many other neurodegenerative disorders, including Alzheimer’s (AD) and Parkinson’s (PD) diseases [7]. In each scenario, the misfolding of a specific protein, that is, the amyloid β-protein (Aβ) for AD, α-synuclein (α-syn) for PD, and the prion protein itself (PrP) for TSEs, can lead to its aggregation and cell-to-cell transfer, forming insoluble deposits or plaques in different regions of the brain (depending on the particular protein under discussion) [8]. To date, there is no available treatment to halt or to delay the neurodegeneration process triggered by one or more of these misfolded and aggregated proteins in the CNS; therefore, these diseases are still invariably fatal. Understanding the molecular basis of protein misfolding and conformational conversion are major priorities in the search for therapeutic strategies that could block or modulate the aggregation process from its very beginning.

The mechanisms that lead a soluble and natively folded protein to adopt an aberrant conformation with a higher tendency to form aggregates depend on the different intermediate structures formed during the folding process, the free energy of these intermediates, the energy barrier between them, and the exposition of hydrophobic surfaces that should be normally buried and solvent-excluded in a functional conformation [9]. Misfolded forms are normally degraded by cell protein quality control mechanisms, but during aging these mechanisms begin to fail, losing or reducing their ability to prevent protein accumulation [10]. Mutations, posttranslational modifications, environmental variations, or interactions with external agents are also factors that can drive protein misfolding and aggregation [11]. PrP is also known as a “promiscuous” protein that can bind to different classes of molecules, including metallic ions, glycosaminoglycans, lipids, and nucleic acids. The biological relevance of most of these interactions is still not clear, but these ligands may participate in the PrP structural conversion and, consequently, in disease progression [12,13,14,15,16,17].

**Figure 1:**
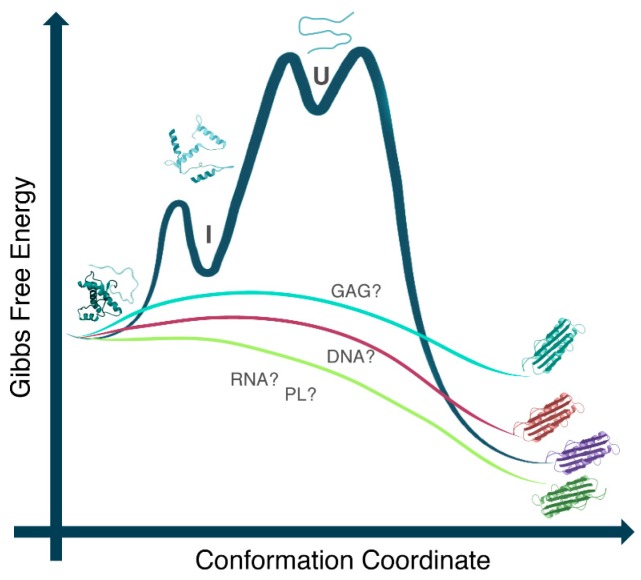
Free energy diagram representing the role of cofactors in prion protein (PrP) conformational conversion. DNA, RNA, phospholipid (PL), and glycosaminoglycan (GAG) candidates may interact with PrP^C^, lowering the energy barrier that prevents its spontaneous conversion to the PrP^Sc^. Different cofactor molecules may stimulate the conversion to the different PrP pathogenic forms and may result in the generation of PrP^Sc^ with varying conformations, providing a possible explanation for the existence of various prion strains. I: intermediate state; U: unfolded state. Reproduced from [9].

Nowadays, the cofactor hypothesis has gained more visibility. It postulates that the presence of an adjuvant factor that interacts with PrP favors its interconversion, aggregation, and infectivity [13,16,18,19,20]. Such a cofactor may act as a catalyst in PrP conversion, lowering the high-energy barrier that prevents the spontaneous conversion of PrP^C^ into PrP^Sc^ (Figure 1). In this context, nucleic acid (NAs) molecules have been ascribed an important role. PrP has been shown to interact with DNAs and RNAs both in vitro and in vivo [21,22,23,24,25,26], indicating their suitable involvement in PrP pathophysiology. Many studies have evaluated the effects of NAs as molecular cofactors for PrP conversion into PrP^Sc^-like species. The in vitro-methodology called SELEX (Systematic Evolution of Ligands by Exponential Enrichment) [27,28] is an interesting tool that has been used to identify and select small oligonucleotides, known as “aptamers” that bind with high affinity and high specificity to the wild-type (full-length) PrP and/or its different domains.

In this review, we will focus on published studies about PrP–NA interactions, the SELEX methodology, the knowledge these bring to the prion field, and the new avenues they offer for the therapy and diagnosis of such devastating diseases. Besides PrP, several other amyloid-forming proteins related to conformational diseases can also bind nucleic acids [25]; therefore, we will also present an overall critical assessment of the aptamer literature in the general field of amyloids, reviewing some relevant SELEX studies against other amyloidogenic proteins, focusing also on the possible drawbacks of this approach regarding aptamer specificity and selectivity against the monomeric or fibrillar forms of these proteins.

## 2. PrP and Nucleic Acids Interactions

The crosstalk between PrP and NAs has captured the attention of the prion research community for the last twenty years. The first study was conducted by Pradip Nandi in 1997 with the human-derived neurotoxic prion peptide (PrP^106–126^) and showed, through fluorescence measurements, the ability of this peptide to bind to a small single-stranded DNA (ssDNA) sequence with micromolar affinity and that this interaction induced a structural change in the DNA molecule [29]. In subsequent publications, Nandi showed that PrP^106–126^ polymerizes into amyloid aggregates in the presence of DNA, either in its circular or in its linearized forms, under experimental conditions where the peptide alone did not polymerize [30]. Wild-type murine recombinant PrP (rPrP) also underwent polymerization in a nucleic acid aqueous solution [31]. In 2001, our group was the first to show the dual role of NAs in changing PrP conformation and aggregation [21]. While PrP interaction with double-stranded DNA (dsDNA) induced the conversion of the full-length recombinant PrP (rPrP) to β-sheet-rich structures and led to rPrP aggregation as revealed by spectroscopic techniques, the same dsDNA oligonucleotides inhibited the aggregation of a PrP hydrophobic domain, the PrP^109–149^ [21]. PrP^109–149^ undergoes prompt aggregation when diluted from a denaturing condition into an aqueous solution; however, the aggregation is completely inhibited in the presence of DNA in a concentration-dependent manner, as verified by light scattering (LS) measurements and through transmission electron microscopy [21,24]. It was also reported that an anti-DNA antibody (OCD4), as well as the gene 5 protein, a DNA-binding protein, is able to catch PrP only from the brain material of prion-infected humans or animals, but they do not capture PrP from non-infected brains [26]. OCD4 seems to present immunoreaction with DNA-associated molecules and this antibody can form a complex with PrP in prion diseases [26]. Moreover, OCD4 detects PrP^Sc^ over ten times more efficiently than an antibody against PrP [26] supporting the proposal that nucleic acids are associated with PrP^Sc^ in vivo. Collectively, these results reinforce the proposal by our group that DNA can participate in PrP misfolding, shifting the equilibrium between PrP^C^ and PrP^Sc^ by reducing protein mobility and favoring protein–protein interactions [21,32].

Following these initial observations, many groups started evaluating the interaction of NAs with both PrP^C^ and PrP^Sc^, unraveling many aspects of this crosstalk. One important area of exploration was to characterize the DNA-binding site on PrP. Studies with different rPrP constructs, mainly using nuclear magnetic resonance (NMR) and small angle X-ray spectroscopy (SAXS) measurements, identified at least two DNA-binding sites in rPrP; one of them in the C-terminal globular domain and the other in the flexible N-terminal region [33,34,35]. In 2012, our group showed that different small dsDNA sequences can individually bind to rPrP, inducing protein aggregation in a supramolecular structure resembling less-ordered amyloid fibrils [24]. We have observed different effects on the structure, stability, and aggregation of rPrP upon interaction with different DNA sequences [24]. The resultant PrP–DNA complex was toxic to murine neuroblastoma (N2a) cell lines, depending on the DNA sequence, but caused no toxicity to human kidney (HK-2) cell lines [24]. Our results suggested that the DNA GC-content is important to dictate the aggregation pattern and the formation of toxic species; in addition, the PrP expression level or some specific factors from the cellular lineage also appeared to be important to mediate PrP toxicity [24]. In 2013, Cavaliere et al. showed that G-quadruplex forming DNA can bind to different forms of PrP with nanomolar affinity and, in accordance with our previous studies, the PrP–DNA interaction led to loss of the secondary structure of both the PrP and the DNA molecule, indicating that there are reciprocal structural changes after DNA binds to PrP [24,36].

PrP–RNA interactions have also been described. The work of the Darlix group showed that PrP has nucleic acid chaperoning activities, similar to nucleocapsid retroviral proteins, indicating that PrP might participate in nucleic acid metabolism (both RNA and DNA) [37,38]. Indeed, rPrP binds different RNAs with high affinity in vitro and in vivo. This interaction promotes the formation of PrP aggregates where PrP becomes resistant to proteinase K (PK) digestion and the RNAs bound to the complex are resistant to ribonuclease (RNase) attack [22,23,39,40]. This interaction is normally abolished when the PrP construct has its N-terminal region truncated (residues 23–~121), as shown by different groups, suggesting that the flexible PrP N-terminal region is important to establish the interaction with RNA [33,40]. In 2003, Deleault et al. described the role of RNA molecules in stimulating prion protein conversion in vitro [23]. The amplification of a protease-resistant PrP^Sc^-like molecule, termed PrP^Res^ (from the PK-resistance property), was evaluated by the in vitro conversion assay based on the protein-misfolding cyclic amplification (PMCA) method [41,42]. PMCA uses diluted prion-infected brain homogenate as a seed to trigger the conversion of PrP^C^ in healthy brain homogenates; the final amplified PrP^Res^ shares many specific characteristics with the scrapie prion propagated in vivo; therefore, it is widely used in prion conversion studies [41]. It was found that RNase inhibits PrP^Res^ amplification in a dose-dependent manner, evidencing that RNA is required for the efficient formation and accumulation of scrapie-like PrP in vitro. Moreover, only the addition of specific RNAs (isolated from mammalian brains) was able to stimulate this conversion reaction [23]. Subsequent work, using only purified and synthetic molecules, revealed that PrP^C^, PrP^Sc^, co-purified lipids, and poly-A RNA can form the minimal set of components necessary to amplify the PrP^Res^ conformation in vitro with the ability to infect normal wild-type hamsters in vivo [18]. The requirement of a negatively charged accessory molecule for the efficient production of infectious prions in vitro (synthetic prions) is in good agreement with the proposed cofactor hypothesis, where endogenous or extracellular factors may participate in prion propagation in vivo. Nevertheless, more studies are required to determine what the exact molecular characteristics of PrP conversion catalysts are and to establish whether one or more cofactors could be considered ‘ideal’ for forming true prions in vitro or to participate in prion pathogenesis in vivo. Our group showed, through several biophysical approaches, that depending on the RNA source—whether from mammalian, yeast, or bacterial cells—the interaction with murine rPrP led to aggregation with different extents. rPrP–RNA interaction led to secondary structural changes in both rPrP, which loses α-helical content, and in the RNA molecule [40]. Finally, only the aggregated species formed upon incubation with RNA extracted from N2a cells were highly toxic to N2a cells in culture [40]. RNA-binding to ovine PrP was also investigated, and the results revealed a likewise PrP conformational shift to a higher β-sheet content, as well as the neurotoxicity of this complex [39]. In accordance with previous work, the PrP N-terminal region seems to be essential to mediate these effects [40,43].

Although PrP^C^ is typically localized anchored at the plasmatic membrane, it has been reported that it can be found in the nucleus of neuronal and endocrine cells and can interact with chromatin [44]. The translocation and deposition of misfolded PrPs in the nucleus of infected cells, where the misfolded PrP was able to interact with chromatin components, has also been shown [45]. Although converging experimental evidence indicates that the endocytic pathway is the principal site of prion conversion [46,47], one might speculate that an abnormal nuclear compartmentalization of PrP may contribute to its encounter with non-native partners that could be involved in prion pathogenesis. Nevertheless, PrP and nucleic acids could crosstalk even along the endocytic pathway, as would be the case of endocytosis of exogenous (or from the membrane) PrP bound to nucleic acid. It would also be possible that cytosolic forms of PrP [46] encounter small NAs in the cytoplasm, triggering conversion. In fact, cytosolic PrP has been shown to induce the formation of large ribonucleoprotein organelles in the N2a cell line [48]. Moreover, PrP^C^ to PrP^Sc^ conversion can also occur on the plasma membrane, being the primary site of conversion when the host is infected with scrapie from external sources [49,50]. In this latter case, a nucleic acid released from a cell or from an exogenous source could encounter PrP^C^/PrP^Sc^ at the membrane.

Altogether, the evidence compiled here concerning PrP–NA interactions strongly suggest that these molecules can be partners in vivo. Both of them can trigger PrP misfolding, leading to its aggregation in vitro, and they can also stimulate PrP^Sc^ conversion and propagation in vivo. Although they are not identical, the misfolded PrPs formed can be toxic to cultured cells depending on the nucleic acid sequence evaluated. We strongly believe that the sequence and structure adopted by the NAs are essential to dictate those effects. More studies about this partnership may be fundamental not only to understand prion function or dysfunction but also for the development of effective therapeutic approaches.

## 3. SELEX Technique and the Aptamer Discovery

**Figure 2:**
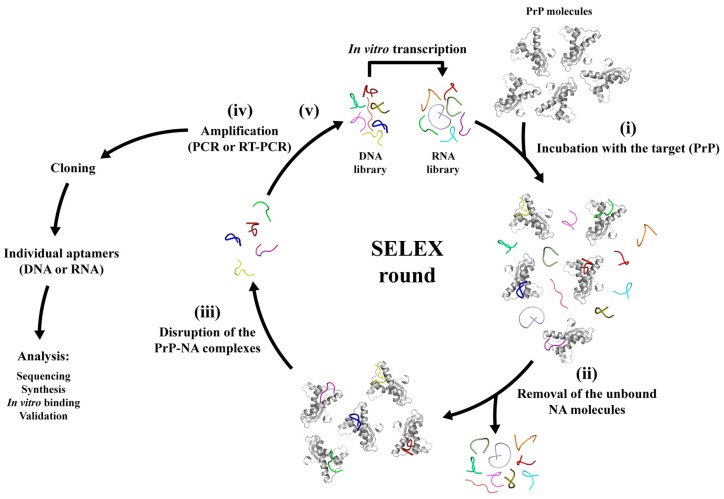
General scheme of the SELEX method using recombinant PrP as the target. A SELEX round consists of the following essential steps: (i) binding after the incubation of a randomly synthesized DNA or RNA library (containing 10^14^–10^16^ different sequences) with the molecular target (full-length recombinant PrP or other PrP constructions); (ii) removal of the non-bound NA species; (iii) elution of NA sequences from the immobilized PrP (either in-column, in ELISA dishes, or other); (iv) amplification of the eluted NA sequences; (v) back to Step (i). This process can be repeated several times to enhance the affinity and specificity of the isolated NA sequences. The final selected NA pool contains the aptamers that have to be further cloned, and individual aptamers have to be sequenced and validated for binding against its target, PrP.

The SELEX technique consists of finding NA ligands with high affinity and specificity against a given target [27,28]. In this review, our target is the PrP. Generally, the core of the selection process consists of the following essential steps: (i) incubation of a randomly synthesized DNA or RNA library (containing 10^14^–10^16^ different oligonucleotides sequences) with the selected target to allow binding; (ii) separation of bound from non-bound species (unbound oligonucleotides are removed by several stringent washing steps of the binding complexes); (iii) elution of the target-bound oligonucleotides with higher salt concentrations; and (iv) amplification of the oligonucleotide bound species by a polymerase chain reaction (PCR). A new enriched pool of selected oligonucleotides is generated by purification of ssDNAs from the PCR products (DNA SELEX) or by in vitro transcription (RNA SELEX). Then, this selected NA pool is used for the next selection round. This process can be repeated several times to enhance the affinity and specificity of the isolated NA sequences. The final selected NA sequences are called aptamers; they have to be cloned and individual aptamers have to be sequenced and validated against its target (Figure 2). The stoichiometry of the target and the NAs can be altered as well as the number of washes, and competitive inhibitors can also be added to the binding buffer to enhance the stringency of the SELEX conditions [51]. A counter-SELEX procedure can also be performed to exclude sequences recognizing other non-interested targets by using similar structural targets, therefore increasing the selectivity of the aptamers [52]. Over the years, many modifications and improvements have been introduced to the classical SELEX methodology in order to decrease the selection time and enhance the binding affinity, which include capillary electrophoresis (CE)-SELEX, automated SELEX, and whole cell SELEX [52]. The cell–SELEX technique is fast, straightforward, and very promising because it can be performed with normal living cells, thus guaranteeing that target proteins on the cell maintain their native conformation and function along the selection procedures [52]. Some aptamers have already been discovered to work against different cancer cells by this method; for example, one of them could specifically recognize leukemia cells [53]. To date, there is only one federally approved aptamer, the Pegaptanib drug, selected against vascular endothelial growth factor (VEGF) to treat the age-related macular degeneration, although there are more than 10 aptamers under different stages of clinical trials for treatment of coagulation, inflammation, cancer, etc. [54]. However, various crucial aspects have delayed the clinical translation of therapeutic aptamers, including their intrinsic physicochemical properties and the lack of safety data.

Aptamers are small single-stranded DNA or RNA nucleotides with a length varying from 20 to 100 bases, flanked by two constant sequences that contain the primer-binding sites [48]. This name comes from the Latin “aptus,” which means “fit”. Single-stranded NAs can fold into a variety of loops, stems, hairpins, quadruplexes, and bugles, among other shapes, to generate a vast range of secondary and tertiary NAs structures [52]. A well-defined three-dimensional structure of these oligonucleotides can specifically recognize and interact with several target molecules, including amino acids [55,56], proteins [57], antibodies [58], or even whole cells [59]. The interaction affinity occurs in the nanomolar to femtomolar range and is established by various intermolecular forces such as hydrogen bonding, van der Waals forces, and base stacking. To date, thousands of aptamers have already been selected against a wide range of target ligands [52]. They are compared to antibodies but have more advantages over them. They are smaller in size, which allows them to reach the cellular target more easily than classical antibodies; they might have more affinity to the specific target; they are easier to synthesize; and they are non-toxic and non-immunogenic [52]. Aptamers are also more thermally stable and can restore their original structure easily and quickly if denatured, while antibodies cannot. These characteristics support the evaluation of aptamers as great therapeutic candidates. Their clinical limitations—such as low stability, because of nucleases’ action in blood, and fast clearance, due to their smaller size—can be easily overcome by further chemical modifications [60]. Site-specific modifications are difficult to make in antibodies, in contrast to aptamers, where chemical modifications can be easily introduced at any desired position in the nucleotide sequence. Because of their ability to bind to proteins and to block their functions, they are also being investigated in the interruption or prevention of misfolded protein accumulation, which is related to many diseases, as discussed here. Moreover, aptamers might differentiate even between isoforms of a given protein, which would aid the understanding of prion function and/or dysfunction, in addition to the diagnostic potential.

## 4. Nucleic Acids Aptamers against PrP

Considering PrP interaction with a large variety of NAs, this protein was proposed as a NA chaperone, although many doubts still persist concerning either its functional or pathological role. Pursuing the main objectives of dissecting PrP–NA interaction, elucidating PrP conformational conversion, identifying specific NAs with which PrPs from different species may interact, and looking for therapeutic and/or diagnostic methods, several studies have applied the SELEX method [61]. With regard to therapeutic perspectives, aptamer(s) binding to PrPs could prevent their conversion and accumulation by stabilizing PrP^C^ or PrP^Sc^, inhibiting PrP^C^-PrP^Sc^ interaction, or blocking PrP^C^ binding to some pathological stimulating cofactor. Specific aptamers for PrPs could also set up new diagnostic tools, since early diagnosis is crucial and will definitely improve the efficacy of any attempt at therapy.

The first aptamer for PrP was selected by Weiss et al. in 1997 using the SELEX method directed against recombinant Syrian hamster full-length prion protein (rPrP^23–231^). The isolated unmodified RNA aptamer did not recognize the C-terminal domain construct (rPrP^90–231^) that lacks almost all the N-terminal residues [33]. They mapped the RNA aptamer-binding site between amino acid residues 23–52 at the PrP N-terminal region. They also suggested that these RNA aptamers may fold into guanine(G)-quartet-containing structural elements that seem to be essential for PrP recognition since the G replacement for uridines (U) in the aptamer sequence abolished its binding to PrP [33]. These individual RNA aptamers interact specially with PrP^C^ from brain homogenates of healthy mice, hamsters, and cattle but did not recognize PrP^Res^ in brain homogenates from prion-infected mice; moreover, the interaction was not observed in PrP knockout mice [33]. The conservation of the specially PrP^C^–RNA interaction in different species provided the first landmark to the development of new diagnostic assays for prion diseases using aptamers.

The same group in 2001 was also the first to show the therapeutic potential of aptamers against prion diseases by selecting the 2′-amino-2′-deoxypyrimidine-modified RNA aptamer (DP7) that was able to reduce PrP^Sc^ accumulation in prion-infected neuroblastoma cells [62]. The RNA chemical modification was made—after using SELEX—as a strategy to enhance the RNA resistance to nucleases. DP7 was highly specific to human PrP^90–129^, a region involved in PrP pathological conversion, and its binding was sustained even for full-length PrP from different species, including humans, mice, and hamsters [62]. One might speculate that blocking this region could supply promise for controlling the conversion. In 2003, Rhie et al. were the first to characterize RNA aptamers that bind preferentially to the infection-related conformations of PrP [63]. The 2′fluoro-RNA aptamer against scrapie-associated fibrils (SAF-93) had a tenfold higher affinity for PrP^Sc^ than for PrP^C^ and inhibited prion propagation in an in vitro conversion assay, highlighting its therapeutic and diagnostic potential [63].

Following these studies, many authors have used the SELEX methodology either against PrP^23–231^ from different species, the PrP C-terminal domains, PrP peptides, or pathogenic-related conformations (Table 1). It has been revealed that aptamer interaction with PrP is sustained even after immobilization and aptamer chemical modification [64]. Briefly, the first DNA aptamers were selected against recombinant human cellular PrP^23–231^ and interacted specially with mammalian PrPs from normal brain homogenates of sheep, calf, piglets, and deer as well as with PrP^C^ expressed in N2a cells [65]. None of them bound to PK-digested prion-infected ScN2a cells, suggesting that these aptamers hold specificity for PrP^C^ [65]. Their binding affinity appeared to be both aptamer sequence- and structure-dependent, with dissociation constants in the micromolar to nanomolar range, in accordance with our work with nucleic-acid ligands selected individually [65]. The work from King et al. selected aptamers against the globular domain (residues 90–231) of hamster PrP, folded into an α-helical-rich native conformation, identifying a thioaptamer (with phosphorothioate modification to enhance their stability against nucleases) with an affinity of 0.58 (+/−0.1) nM for hamster PrP [66]. Lower affinities for bovine (Bo) and human (Hu) PrP were found, suggesting some specificity where the interaction is dependent on the primary structure of PrP [66]. A control oligonucleotide with the same length and a scrambled consensus sequence could not differentiate among the three PrP sequences, and control oligonucleotides encompassing non-selected sequences bound to PrP at a sequence-independent DNA-binding site with much lower affinities [66]. The results confirm that the high-affinity binding of thioaptamers to PrP depends on backbone modifications, oligonucleotide sequence, and PrP sequence [66].

As well as RNA aptamers, the ssDNA thioaptamers designed by Kocisko et al., were able to bind PrP^C^ on live cells, to be cell-internalized and potently inhibit PrP^Res^ accumulation in infected-cultured cells [67]. Interestingly enough, prophylactic treatments with these modified oligonucleotides tripled scrapie survival periods in mice [67]. A prolonged survival time was also observed when these phosphorothioate aptamers were previously mixed with the infectious brain inoculum [67]. The potent anti-scrapie activity of these modified nucleic acids represents a new class of drugs that hold promises for the treatment of prion diseases [67,68]. Mashima et al. in 2009 provided the first report, showing the high-resolution structure of an RNA aptamer (R12) against isolated domains of the bovine PrP by NMR. The GGAGGAGGAGGA sequence from R12 aptamer forms an intramolecular parallel G-quadruplex structure [69]. G-quadruplexes are formed by G-rich sequences and are built around tetrads of hydrogen-bonded guanine bases (Hoogsteen base pair). Two or more G-tetrads can stack on top of each other to form the structure, and the quadruplex is stabilized by a cation, especially potassium [70]. Two R12 quadruplexes form a dimer through intermolecular hexad–hexad stacking [69]. Most of the RNA aptamers obtained by this group contain GGA tandem repeats and bind both rPrP^C^ and the beta-form of PrP with high affinities [71]. The DNA counterpart aptamer (D12) can also bind to PrP, but the affinity is weaker for both cellular PrP and its β-form [36,69]. The GGA tandem repeats form peculiar quadruplex structures that appear to be critical for the higher affinities and recognition of PrP. This tight binding is expected to stabilize PrP^C^ or block its interconversion and to thereby prevent the onset of prion diseases.

NMR measurements also provide the first high-resolution 3D-structure of the complex formed with N-terminal PrP peptides (P1 and P16) and the R12 aptamer [72]. The G-quadruplex structured RNA is preserved even after interaction with PrP. The RNA forms a dimer where each monomer simultaneously binds to two portions of the PrP^C^ N-terminal region, which can explain the strong binding, where electrostatic and stacking interactions drive the affinity of each portion [72]. Additionally, the authors demonstrate that the driving force for the binding between R12 and P16 (a PrP peptide) is a robust gain of water entropy, and the energy decrease driven by attractive interactions between R12 and P16 is compensated by the energetic dehydration effect after binding or vice-versa. The interaction of the complex occurs via stacking of flat moieties, via electrostatic interactions, including specific hydrogen bonding, and through molecular geometry complementarity [73].

One should not forget that an appropriate geometrical correspondence of hydrogen bond donors and acceptors may allow more stable complexes to be formed, but it is mainly due to stacking interactions that significant stabilization occurs [74]. Moreover, R12 was shown to reduce the accumulation of PrP^Sc^ levels in scrapie-infected neuronal cells, demonstrating its great therapeutic potential [65]. Remarkably, G-quadruplex forming NAs were shown to change the PrP^C^ structure after binding, so that they may actually lower the free energy barrier between the two conformers and therefore prompt the conversion process [36]. As one of the strongest PrP binders, more attention should be given to these peculiar NA structures. In fact, many NA ligands directed against PrP discussed here might form G-quadruplex structures, as they contain at least four GG repeats in their sequence that could form quadruplexes after dimerization. The PrP messenger RNA (mRNA) itself has sequences with the propensity to form G-quadruplex depending on environmental conditions such as fluctuations in potassium levels [75]. One cannot rule out the possibility that PrP interaction with its own mRNA might be involved in their physiological or pathological pathways.

## 5. Aptamers against Other Amyloidogenic Proteins

The prion protein has been understood as the etiological agent of TSEs amyloidosis, but nowadays the term “prion” has evolved to describe a phenomenon in molecular biology that is more ubiquitous than previously thought and that is shared by other prion-like proteins, especially the ones that can aggregate into amyloid fibrils through a highly ordered mechanism. Unrelated proteins in their sequence or structure can form amyloid aggregates that possess a common cross-architecture with a β-sheet enriched core; it appears that the amyloid fibril formation is the result of an intrinsic, conservative, and generic process of proteins, and amyloids formed by the same protein sequence can still be found with different structural and phenotypic properties [7]. Evidence indicates that amyloid fibrils share specific structural characteristics and aggregate morphologies; however, some structural polymorphisms between amyloids can be found either in vivo or in vitro [83]. Although our review focuses on PrP interactions with nucleic acids, we find it useful to also provide an overall critical consideration regarding aptamer interactions with other prion-like amyloid proteins.

In this context, RNA aptamers were selected by SELEX against the wild-type bovine PrP (bPrP); the recognition was shown to occur mainly through the PrP N-terminal (25–131) region, as expected and confirmed by other researchers [40,71]. However, and interestingly, those same aptamers also bound to bPrP in the β-conformation (bPrP-β), which resembles the amyloid core, but with a tenfold lower interaction affinity [71]. Thus, it led the group to conclude that the selected aptamers bind with high affinity to both native PrP and the amyloid-like PrP conformation. The transition from the native α-form to a β-form was achieved, and it occurs briefly in the presence of phospholipid micelle solutions at pH 5.0 [71]. One question raised is this: How did an aptamer selected against a non-amyloid prion form recognize the β-conformation? The same bPrP-β formation protocol was applicable to human, cow, elk, pig, dog, and mouse PrP, even in wild-type or truncated PrP forms, and it showed that part of the flexible domain encompassing the 105–120 region must be present for the generation of bPrP-β [71]. Although that region is normally unfolded in the native PrP, it can also undergo dynamic structural shifts. This region is normally positively charged but also contains hydrophobic amino acids, both important for NA-binding. One might speculate that the epitope recognition motif found in the 105–120 PrP region can be found both in the native or in the amyloid PrP conformation; the aptamer interaction is established with high affinity for the two forms (bPrP and bPrP-β), but with a significant difference between them, probably because, for the latter, the new structure potentially adopted by the 105–120 region might change the dissociation constant value. Besides, the results showed that the high affinity for bPrP-β by the selected aptamer is abolished at a high salt concentration (1M NaCl); this behavior is not observed for the native bPrP, which maintains approximately the same interaction affinity for the same selected aptamer in equal experimental conditions [71]. This suggests that this aptamer interaction with the amyloid PrP form occurs mainly through electrostatic interactions, but for the native PrP, electrostatic interactions may bring the two partners in proximity, allowing them to establish more specific intermolecular forces (hydrophobic and base stacking), resulting in a higher affinity for the native PrP. In addition, when minimizing the aptamers’ length, they lose their affinity for the bPrP-β (30-fold less) in comparison to bPrP, demonstrating that a differential aptamer specificity indeed exists for the two PrP conformations [71]. We thus suggest the use of non-aggregated PrP forms as competitors to improve selection and binding-ability to β-forms of PrP.

Besides PrP, we will also discuss briefly some studies about other amyloidogenic proteins related to diseases, such as Aβ protein and β-2-microgobulin (β2m), regarding their interaction with aptamers. RNA aptamers were selected against Alzheimer’s amyloid Aβ peptide (1–40), and, apparently, the selection targeting a non-amyloid Aβ conformation led to the selection of aptamers that recognized the β-sheet-rich fibrils of Aβ [84]. Aβ (1–40) presents a hydrophobic domain that aggregates easily, especially in amyloid fibrils, but the experimental condition of Aβ (1–40) immobilization for the SELEX process in this particular study did not guarantee that the monomeric conformation of Aβ (1–40), that is, the SELEX target, was maintained in-column [84]. Aβ (1–40) could possibly aggregate in-column and/or will also be found in the trimeric or tetrameric form. It cannot be ruled out that those oligomeric forms present a β-sheet core enriched enough to be considered a pre-amyloid aggregate, which could explain the positive selection of these aptamers to the mature amyloid fibrils as well. Preformed aggregates induce fast aggregation of amyloidogenic proteins, resulting in poor experimental reproducibility [85], and are not desirable in aptamer selection for non-aggregated, non-fibrillar forms of the respective proteins. The interaction of Aβ (1–40) fibrils with these particular RNA aptamers can be easily detected through electron microscopy; the aptamer was labeled with colloidal gold that stained as black dots along the amyloid-fibrils, showing that the interaction occurs in specific regions of the fibril with an apparently specific distribution pattern [84]. Again, the addition of experimental controls like other amyloid fibrils or completely non-aggregated proteins is of interest to refine the executed assay.

Subsequently, the study performed by Rahimi et al. provided a step forward, exploiting aptamers’ interaction with amyloid fibrils formed by distinct proteins [86]. The results showed that an aptamer selection targeting a non-fibrillar Aβ preparation led to a selection of aptamers that recognized fibrils of Aβ and fibrils of other amyloidogenic proteins [86]. Although we consider this work strongly relevant, some considerations can be explored for relevant discussion and speculation of other possibilities regarding the experimental evidences and conditions; some of them were also raised by the authors [87]. One of the aptamer selection targets was the cross-linked trimeric form of the Aβ protein using the filter-binding SELEX assay. The authors selected the Aβ trimeric conformation by direct purification from the SDS-PAGE and stored it for approximately twenty-four hours in a solution containing traces of SDS before the final procedure, which consisted of long-term dialysis to remove impurities until the sample was ready for the SELEX’s first round [88]. These procedures suggest that traces of SDS may have been present in the sample and/or the extensive dialysis duration might have accelerated Aβ self-aggregation, enhancing the β-sheet content and/or favoring protein–protein interaction and aggregation, as verified for other proteins [89,90]. In addition, a nitrocellulose filter-binding assay is not the most suitable way to retain low-molecular-weight proteins such as Aβ (1–40), due to the poor retention of the peptide on the filter. This approach might result in the apparent unexpected lack of interaction between the aptamers selected against this specific Aβ assembly (in the trimeric expected conformation), with the same expected assembly verified through a filter-binding assay [86]. The aptamers were probably not selected against a homogeneous non-aggregated Aβ-form; we therefore believe the SELEX target was a common cross-beta structure present both in the trimeric form, oligomers and in the amyloid fibrils. Moreover, the evidence that two aptamers have different binding affinities between fibrils formed with the Aβ (1–40) and Aβ (1–42) reveals that there must be some specificity governing the aptamer interaction [84]. The reactivity of aptamers against Aβ (1–42) fibrils was somewhat lower than their reactivity with Aβ (1–40) fibrils, again suggesting moderate specificity for Aβ (1–40) [84]. Given that observation, there are minor differences between Aβ (1–40) and Aβ (1–42) that come from a dissimilar enzymatic cleavage site of the amyloid precursor protein (APP), and there are still significant differences upon binding to the selected RNA aptamers, strongly suggesting some specificity, which we are still looking for. Remarkably, these same aptamers recognize fibrils of other amyloidogenic proteins, including insulin, islet amyloid polypeptide, calcitonin, lysozyme, and PrP^106–126^, but with significant differences between some fibrils that might correlate with residual specificity [86].

Similar results were obtained in other work, where aptamers were selected against fibrils of β2-microglobulin (β2-m) or against monomeric β2-m at low pH [91]. The aptamers bind with high affinity to β2-m fibrils with different morphologies formed under different conditions in vitro, as well as to amyloid fibrils isolated from tissues of β2-m-related amyloidosis patients, demonstrating that they can detect conserved epitopes between different fibrillar assemblies of β2-m, including those formed in vivo. At this time, the group’s data demonstrate that the selections generated aptamers able to bind with high affinity to all three forms of β2-m, including two distinct fibrils and the low pH monomeric form [91]. The validation of the interaction was performed through surface plasmon resonance (SPR), a more suitable and refined method for this approach than dot-blot only [91]. These results suggest that the β2-m fibrils share at least one epitope in common with the monomeric β2-m, and the higher affinity for the fibrillar form might be due to more epitopes being available and the greater ease of interacting with these aptamers in the macromolecular structure of the fibril. The aptamers also reacted with some (but not all) other amyloid fibrils, either generated in vitro or isolated from ex vivo sources; but for these other proteins, none of the aptamers were able to bind to native monomers, confirming that the epitopes being recognized are fibril specific [91]. Thus, the native folded species seem not to share epitopes in common with the fibrillar and pre-fibrillar states. SPR measurements also showed that there are large signal differences between the naive SELEX RNA pool and the final selected individual aptamers against the target, confirming that the SPR responses seen are due to specific binding and not to inherent affinity of the oligonucleotides for fibrillar amyloid structures [91]. The same behavior was reported for certain antibodies, which could recognize conformational epitopes in Aβ assemblies and interact with similar assemblies of other amyloid-forming proteins [92,93,94,95]; some antibodies were raised against oligomers but reacted with both oligomeric and fibrillar assemblies [96,97,98].

One must conclude, based on these observations, that, although amyloid fibrils have many common structural properties, they also have features that are unique to individual fibril types. Some amyloids may hinder more structural differences than others, and because of these structural polymorphisms, aptamers selected against a specific amyloidogenic protein can interact with other unrelated amyloid-forming proteins with similar or significantly different affinities, depending on the amyloid protein aggregated structure. There were also cases where these aptamers have not at all recognized amyloid fibrils from other amyloidogenic proteins, such as apomyoglobin, Aβ (1–40), or transthyretin, but significant binding was observed to fibrils formed from lysozyme [91]. The aptamer binding to lysozyme fibrils cannot be the cause of nonspecific interactions, as evidenced by the inability of the aptamer to bind to native monomeric lysozymes together with the observation that the naive RNA SELEX pool binds relatively weakly to the lysozyme fibrils under these conditions [91]. Although these aptamers recognize an epitope present in different amyloid fibrils, the epitope for each aptamer must be either distinct or differentially accessible between different amyloids. Understanding the structural molecular basis of why some aptamers and antibodies raised against monomeric proteins can recognize either the oligomeric forms or the amyloid polymeric architecture requires an ongoing investigation, but will definitely improve the discussion about their potential in the pharmaceutical and biotechnology fields.

## 6. Conclusions and Perspectives

Over the last twenty years, NAs have been proposed as potential cofactors that can bind to different disease-related proteins and can trigger their misfolding and aggregation processes [9,99]. Protein interactions with NAs are governed by several molecular forces, including hydrogen bonding mediated by aqueous solvent, electrostatic, hydrophobic, and stacking interactions [52]. Because of the many structural motifs existing both in proteins and NAs, as well as the variations in the nucleotide sequences, it is very difficult to characterize a single model for protein–NA interactions. Hydrophobic interactions seem to be more efficient than charge effects for driving protein aggregation [100]. However, both factors (hydrophobic and charge effects) can be critical and determinant for protein aggregation and need to be considered for understanding the process in vivo and the role of amino acid composition, sequence, and substitutions in protein misfolding diseases and protein design [100,101].

The knowledge acquired from the PrP studies discussed here permitted us to map the NA-binding sites on PrP. This interaction involves at least three different binding sites: two of them localized at the N-terminal flexible region and the other in the structured globular C-terminal domain. Apparently, the two lysine clusters in the N-terminal domain, encompassing residues 23–52 and 101–110, are involved in all non-specific NA interactions, since this is a positively charged region with enough flexibility to bind DNAs, RNAs, and even heparin molecules, mainly through electrostatic interactions with the sugar-phosphate backbone [35,63]. Through NMR studies, residues encompassing the lysine cluster were shown to mediate the interaction with DNA [34] or RNA [72]. Although contributions from hydrophobic interactions appear to be more important than those involving charge interactions, the influence of charge factors on protein aggregation must not be underestimated [100]. Structural data show that the PrP globular domain in the normal conformation can interact with DNA but not with RNA [35,40]. However, conformational changes in the C-terminal domain, especially in its transition to beta forms, can expose structural or sequence motifs that are able to bind even RNA aptamers through more specific interactions than those established with the N-terminal region [63].

Although many efforts have been made to find the sequence specificity governing PrP-NA interactions, no consensus has yet been found. Partial consensus sequences are clearly present, confirming that selection had occurred, but there has been no obviously dominant epitope-binding consensus. Comparison of the aptamers sequences reported elsewhere for either anti-PrP or anti-Aβ (1–40) or anti-β2-m aptamers did not show significant sequence motif matches, suggesting that the aptamers raised are specific to their selection targets. Some structural features can be highlighted, such as the G-forming quadruplex in many DNA and RNA ligands that provides tight bonding; however, other structural motifs, such as hairpins and the double helix, have also been described and proven to have a high affinity for PrPs [24,36]. Through NMR studies, it appears that the geometric characteristics (overall shapes, sizes, and detailed polyatomic structures) of the molecules are the most important factors governing PrP recognition [73]. The strong diversity between PrP nucleic acid ligands should not rule out the existence of some specificity, once many modifications in the NA molecule can alter its PrP-binding affinity and can trigger different changes on PrP properties [24,33].

NA molecules might play a dual role in prion biology, either by triggering PrP conversion and aggregation or by preventing them. Understanding this intriguing partnership could be critical to explaining how prion diseases arise, and to developing effective diagnostic and therapeutic methodologies. In terms of the pathological aspect, NA-binding to PrP could lead to reciprocal conformational changes, altering both the PrP and NA structure and promoting distinct modes of polymerization depending on the NA source. Possibly the charge neutralization of the positively charged PrP N-terminal domain after NA binding favors the association of PrP molecules, which might explain the immediate NA-induced PrP aggregation [102].

What remains to be elucidated is whether these interactions are relevant in vivo, regarding either the pathology or biology of prions. Although RNA molecules were found to be associated with plaques in the brains of AD-diseased patients [103,104,105], no direct evidence of in vivo association of specific nucleic acid sequences with PrP scrapie in affected humans has been found yet. Nevertheless, the work of Manuelidis’ group showed that circular DNAs could be co-purified along with infectivity in 22L-infected cell lines, in hamster 263K scrapie-infected brain samples, and in FU-CJD infected mouse brain [106]. Additionally, the same group showed that prion infectivity was retained when PrP was digested; in contrast, when different prion strains were treated with nucleases, infectivity (prion titer) was substantially reduced [107]. In addition, PrP-NA interaction was shown to be fundamental in generating synthetic scrapie prions; free small RNAs, extracted from highly infectious scrapie-associated fibrils (SAFs) and incubated with PrP^C^, were shown to promote PrP^C^–PrP^Sc^ conversion with the acquisition of infectivity, inducing prion disease in wild-type healthy Syrian hamsters [108]. These results indicate that nucleic acids are essential for prion infectivity and might be involved in prion pathogenesis.

Altogether, these studies show that NAs are potential PrP cofactors able to catalyze the formation of PrP^Sc^ in vivo. The “NA cofactor hypothesis” initially proposed by our group and reinforced by other contemporaneous studies does not necessarily refute the commonly accepted “protein-only hypothesis”, where PrP is the solely proteinaceous infectious agent. In fact, we otherwise add to this vision the suitable participation of molecules that could facilitate protein aggregation and the formation of infectious conformations that could, even alone, template the conversion of normal PrPs into abnormal conformations, thus leading to prion disease progression.

Protein folding and protein aggregation are dynamic and competitive events constantly fighting inside the cell and driven by the same molecular forces, which explains why these processes are well controlled and balanced by the protein quality control of the cell machinery [109]. The process is so complex that an increasing number of proteins with the same amino acid sequence were shown to adopt, under native conditions, various folded conformations that coexist in dynamic equilibrium [109]. Especially for the amyloid aggregation pathway, there are many precursor species along the way to fibril maturation: amyloid seeds, oligomeric forms, prefibrillar, and fibrillar states [7]. Therefore, amyloid aggregation is also a dynamic and potentially reversible process where different species may be present even after fibril formation. Regarding aptamer selection against amyloid prions, the characterization of aptamers is particularly important, because the natural affinity of oligonucleotides for fibrillar amyloid structures potentially hinders the development of aptamers that are specific for non-fibrillar amyloid proteins under physiological conditions [86]. Particular studies discussed in this review describe the selection of nucleic acids that inherently bind fibrillar or β-sheet-rich structures of amyloid proteins. This tendency must be more deeply explored. In addition, it is important to determine the size distribution profile of the amyloid aggregates as well as the morphology of each species that can coexist in the aggregation protocols used in those studies, making it more difficult to guarantee which exact species are exposing the exact epitope that led to the aptamer selection. In general, aptamers selected against amyloidogenic proteins recognize a structural motif, probably the backbone of the proteins in a cross-β structure that is common to the fibrillar state of these proteins. Nucleic acid reactivity clearly depends on the protein assembly state and to some extent on the protein sequence. Based on the idea that the amyloid fold is ancient and may have co-evolved with RNAs [110,111], it is plausible to propose that such proteins present a general nucleic acid binding property resulting from this evolution process. NA-binding can thus result in ribonucleoprotein complexes that possess important cellular functions, for instance, being related to functional amyloids [112] or to amyloidogenic diseases [99]. Accordingly, it is expected that amyloids and amyloid-forming proteins will present promiscuous RNA- (or even DNA)-binding characteristics.

Developing effective therapies against prion diseases and other amyloidosis remains a hard challenge. Together with the therapeutic potential of aptamers against PrP, ligands able to bind and stabilize the native state of an amyloidogenic protein provide one such potential strategy for controlling protein accumulation and the disease progression of many neurodegenerative disorders including Alzheimer’s and Parkinson’s diseases [51]. Efforts to generate aptamers that would specifically recognize oligomeric pre-amyloid species have also been a challenge, likely due to the dynamic nature of the oligomers preventing long-lasting NA–oligomer interactions. This inherent, apparently sequence-independent, affinity of oligonucleotides may have led to the generation of fibril-cross-reactive aptamers in studies aiming to generate aptamers for non-fibrillar amyloidogenic proteins. Recently, Takahashi et al. have selected aptamers against an oligomeric model of Aβ (1–40) and demonstrated an interaction with monomeric Aβ with micromolar affinity [112]. However, the cross-reactivity of these aptamers with fibrillar Aβ (1–40) or with other fibrillar amyloidogenic proteins was not determined [113]. In addition, data in the literature indicate that aptamers can also be used to detect early β-sheet formation more sensitively than the common thioflavin-T (ThT) amyloid dye [86]. Thus, these aptamers could be highly efficient detection tools of β-sheet formation in histopathological and in biophysical studies in vitro.

Overall, if aptamers are to be obtained for diagnostic and therapeutic approaches in amyloid diseases, the use of such a selective powerful tool is yet to be achieved in this field. Additional experiments to generate devoted and specific aptamers for prefibrillar assemblies (including monomers and oligomers) will have to deal with the apparent inherent affinity of oligonucleotides for fibrillar structures. Nevertheless, small differences in specificity and affinity of aptamers for amyloid and monomeric proteins may indeed allow their application in diagnosis or therapy.

In the context of the biology and pathology of prion proteins as well as in other protein-misfolding diseases, it would be valuable for those who would like to continue researching aptamers and their applications to find the ideal aptamer against therapeutic targets of the future, specific enough to warrant their use as recognition tools or therapeutics. Maybe the literature is being too optimistic in this regard, but we cannot forget those are still invariably fatal diseases where researchers are avidly waiting for a new drug discovery to treat or cure illnesses for both humans and animals. Several nucleic acids, especially the aptamers for PrP, have been shown to bind to PrP^C^ or PrP^Sc^ and to interfere with PrP^Sc^ biogenesis, providing a new class of promising molecules that could be used for the treatment of prion diseases. Even if they bind monomeric PrP^C^ to some extent, the benefit of preventing conversion into PrP^Sc^ would surpass the drawback of lack of specificity. Some of the investigated nucleic acids have shown therapeutic efficacy in infected mice models by tripling their survival time [67], but to our knowledge none of them have proceeded to clinical studies so far. Alternative attempts based on antibody therapy also have potential [114]; however, the stimulation of the autoimmune system presents challenges to further developments in this area [114].

Unfortunately, there is no therapy to treat or prevent prion diseases. Most of the lead compounds found in the drug screening for anti-scrapie activity lack efficacy (possibly due to prion strain specificity), and have poor pharmacokinetic profiles, such as high toxicity and/or an inability to efficiently cross the blood–brain barrier (BBB) [115]. In fact, the aptamer pharmacokinetic profile is especially relevant for neurodegenerative disorders, pushing the development of strategies towards crossing the BBB, as it is unlikely that they can easily enter the brain. Nevertheless, aptamers may surpass this barrier via pinocytosis, transcytosis, channel, and/or receptors to their uptake [116]. Additionally, quadruplex-structured aptamers may cross the BBB through binding to nucleolin via micropinocytosis [117]. Using a nicely executed in vivo selection protocol, Cheng and collaborators selected aptamers that permeated the brain after peripheral injection of the library in wild-type mice [118]. Fortunately, aptamers are molecules that can be easily modified to overcome their clinical limitations: for example, nanoparticle-encapsulated aptamers were reported to cross the BBB, and delivery of liposome-based aptamers was well tolerated in clinical trials [119]. We still do not have the safety profile of these molecules, although they are expected to be non-toxic and non-immunogenic [52].

In conclusion, NA aptamers can distinguish normal and abnormal conformations of PrP, representing the first reagents able to identify PrP pathological conformations from multiple host species. They can even differentiate prion strains and can be used to detect infectious prions in blood samples, which cannot be accomplished using conventional diagnostic tools that rely on antibody-based detection methods. The hard challenge of prion disease diagnosis before the symptomatic stage is how to discriminate and detect the minute quantity of disease-associated prion protein isoform (PrP^Res^) sensitively and selectivity in complex biological samples, from plasma to brain homogenate. The development of a dual-aptamer strategy for diagnostic tools began with an investigation of the advantages of aptamers, the great separation ability of magnetic microparticles (MMPs), and the high fluorescence emission features of quantum dots (QDs) [120]. Two aptamers were coupled to the surfaces of MMPs and QDs, respectively, which then could be co-associated through the specific interaction of the two aptamers with their two corresponding different PrP epitopes, forming an aptasensor platform [120]. Moreover, aptamers can enrich a target, for example, the PrP molecule, from biological fluids; in this context, RNA aptamers have been successfully utilized for the concentration of PrP^C^ and PrP^res^ taken from serum, urine, and brain homogenate [121]. There is also an interesting proposal for PrP^Sc^-enrichment, using PrP^C^-specific aptamers to capture normal prions from biological samples, which could be used as a diagnostic tool in double ligand assay systems and other aptasensors [65]. There is an urgent necessity to develop more sensitive and more efficient assays to detect the pathological forms of PrP in pre-symptomatic screening of tissue, blood, or other body fluids. Based on these promising studies, NA aptamers appear to be good candidates to reach this goal. Although many aptamers have been identified against PrP, with great potential for use in diagnostic tools, the community is still relying on antibody-based detection methods. Among the limiting factors that make aptamers especially promising is the sensitivity of detection. Thus, many efforts are now being made to build aptasensing platforms based on electrochemical or dual-signal systems to develop highly sensitive prion assays [122,123]. This new class of molecules thus has great potential.

## Figures and Tables

**Table 1:** Binding characteristics of mammalian PrPs and nucleic acids.

Author, Year (Ref.)	Nucleic Acid Type	K_D_^1^ (nM)	Binding Assay	PrP SELEX Target	PrPs Recognized	PrP Binding Region(s)
Weiss, 1997 [33]	RNA-aptamer	ND	Gel-shift of labeled aptamer	Hamster rPrP^23−231^	Mouse, hamster, cow (PrP in brain homogenates)	(23–36)
Nandi, 1997 [29,30,31]	Plasmid DNA	250	Fluorescent dye displacement	NS	Human rPrP^106–126^ and rPrP^23–231^	ND
Cordeiro, 2001 [21]	Short dsDNAs	25	Fluorescence polarization	NS	Murine rPrP^23–231^	N-terminal and C-terminal domains
Gabus, 2001 [76]	HIV-1 LTR DNA (1000 bp)	ND	Gel-shift assay	NS	Human rPrP^23–231 or 23–144^	N-terminal
Gabus, 2001 [37]	HIV-1 5’-leader RNA (415 nt)	ND	Gel shift assay	NS	Human rPrP^23–231^; Ovine rPrP^25–234^	N-terminal
Proske, 2002 [62]	RNA-aptamer	100	Filter-binding assay	Human PrP^90−129^	Hamster, mouse or human rPrP	(90–129)
Adler, 2003 [22]	Small, highly structured RNAs	3.8	Gel shift, filter-binding assay	NS	Human rPrP, PrP from brain homogenates of mouse, rat and hamster	N-terminal domain
Rhie, 2003 [63]	RNA-aptamer	16	Homologous competition binding assay	SAF material from infected brain homogenates	Bovine rPrP in b-oligomeric or a-helical form, PK-untreated SAF, PK-treated SAF	N-terminal and SAF conformation-specific site in (110–230)
Sayer, 2004 [77]	RNA-aptamer	6.8	Equilibrium binding	Bovine rPrP^23−230^	Bovine rPrP	ND
Sekiya, 2005 [78]	RNA-aptamer	ND	ND	Murine rPrP^23−231^ and murine SAF infected material	Murine rPrP^23–231^ and mouse SAF	(23–108) of Murine rPrP and mouse SAF
Sekiya, 2006 [79]	RNA-aptamer	5.6	Filter-binding assay	Murine rPrP^23–231^ with competitive selection	Murine rPrP^23–231^, Bovine rPrP, Mouse PrP in brain homogenate	(23–108) and (23–88)
Mercey, 2006 [35]	RNA-aptamer	15	Surface plasmon resonance, filter-binding assay	Ovine PrP^23–231^ with mutations associated with disease	Ovine rPrP(ARR, VRQ, AHQ, ARQ), Murine rPrP, Bovine rPrP	(25–34) and (101–110)
Lima, 2006 [34]	Short dsDNAs	90	Fluorescence polarization and SAXS	NS	Murine rPrP^23–231^	N-terminal and C-terminal
Takemura, 2006 [65]	DNA-aptamer	16	End-point titration method in microplate, gel-shift, and dot-blot assays	Human rPrP^23−231^	Murine rPrP^23–231^, PrP from brain homogenates of sheep, calves, pigs, deer, PK-untreated PrP from ScN2a cells	(23–89)
Ogasawara, 2007 [80]	DNA-aptamer	100	Surface plasmon resonance, dot-blot, competitive selection and fluorescence measurements	Murine rPrP^23−231^	Murine rPrP^23–231^	ND
Murakami, 2008 [71]	RNA-aptamer	31	Surface plasmon resonance	Bovine PrP^23−231^	Bovine rPrP^23–231^	(125–231)
Bibby, 2008 [81]	DNA-aptamer	18	Saturation binding using PrP-coated Ni-NTA beads	Ni-NTA beads coated Murine PrP^90−231^	Murine rPrP^90–231^, Ovine rPrP and Human rPrP^90–231^	(90–230)
Mashima, 2009 [69]	G4 RNA-aptamer	8.5	Northwestern blotting assay	Bovine PrP^23−231^	Bovine PrP^C^	(25–34) and (110–118)
	G4 RNA-aptamer	280			Amyloidogenic bovine PrP-β	ND
	G4 DNA-aptamer	85			Bovine PrP^C^	(25–34) and (110–118)
	G4 DNA-aptamer	>280			Amyloidogenic Bovine PrP-β	ND
Wang, 2011 [82]	DNA-aptamer biosensor immobilized	22	Surface plasmon resonance	PrP^Sc^ from brain tissues of scrapie-infected animals with counter-selection with PrP^C^	Pathological isoforms of PrP from distinct species	ND
Macedo, 2012 [24]	Small dsDNAs	ND	Fluorescence measurements	NS	murine rPrP^23–231^ and rPrP^109–149^	N-terminal and C-terminal domains
Cavaliere, 2013 [36]	G4 DNA-aptamer	62	Surface plasmon resonance and Isothermal Titration Calorimetry (ITC)	Ovine rPrP-23–231	Ovine rPrP^23–24^	23-134
	G4 RNA-aptamer	75			Ovine rPrP^23–24^	
	G4 DNA-aptamer	300			Amyloidogenic Ovine PrP-β	ND
	G4 RNA-aptamer	400			Amyloidogenic Ovine PrP-β	

We chose the KD (dissociation constant) value of the best interaction when several aptamers were described by the same study. When many types of PrP were investigated in binding assays, the PrP species or fragment with the lowest KD value is the first shown. NS: non-SELEX (NA sequences found individually); ND: non-determined.
